# Evaluation of Cyclic Peptide Inhibitors of the Grb7 Breast Cancer Target: Small Change in Cargo Results in Large Change in Cellular Activity

**DOI:** 10.3390/molecules24203739

**Published:** 2019-10-17

**Authors:** Jianrong Sang, Ketav Kulkarni, Gabrielle M. Watson, Xiuquan Ma, David J. Craik, Sónia T. Henriques, Aaron G. Poth, Aurélie H. Benfield, Jacqueline A. Wilce

**Affiliations:** 1 Department of Physiology, School of Medicine, Jiangsu University, 301 Xuefu Road, Zhenjiang 212013, Jiangsu, China; sangjianrong@ujs.edu.cn; 2 Biomedicine Discovery Institute, Department of Biochemistry and Molecular Biology, Monash University, Wellington Road, Clayton 3800, Australia; ketav.kulkarni@monash.edu (K.K.); gabrielle.watson@monash.edu (G.M.W.); hugh.ma@monash.edu (X.M.); 3 Institute for Molecular Bioscience, The University of Queensland, Brisbane 4072, Australia; d.craik@imb.uq.edu.au (D.J.C.); sonia.henriques@qut.edu.au (S.T.H.); a.poth@imb.uq.edu.au (A.G.P.); aurelie.benfield@qut.edu.au (A.H.B.); 4 School of Biomedical Sciences, Institute of Health & Biomedical Innovation, Queensland University of Technology, Translational Research Institute, Brisbane 4102, Australia

**Keywords:** bicyclic peptide, Grb7, SH2-domain, inhibitor binding, cell penetrating peptide, cancer cell migration, proliferation, invasion, lipid bilayer interactions, mass spectrometry quantification

## Abstract

Grb7 is an adapter protein, overexpressed in HER2+ve breast and other cancers, and identified as a therapeutic target. Grb7 promotes both proliferative and migratory cellular pathways through interaction of its SH2 domain with upstream binding partners including HER2, SHC, and FAK. Here we present the evaluation of a series of monocyclic and bicyclic peptide inhibitors that have been developed to specifically and potently target the Grb7 SH2-domain. All peptides tested were found to inhibit signaling in both ERK and AKT pathways in SKBR-3 and MDA-MB-231 cell lines. Proliferation, migration, and invasion assays revealed, however, that the second-generation bicyclic peptides were not more bioactive than the first generation G7-18NATE peptide, despite their higher in vitro affinity for the target. This was found not to be due to steric hindrance by the cell-permeability tag, as ascertained by ITC, but to differences in the ability of the bicyclic peptides to interact with and penetrate cellular membranes, as determined using SPR and mass spectrometry. These studies reveal that just small differences to amino acid composition can greatly impact the effectiveness of peptide inhibitors to their intracellular target and demonstrate that G7-18NATE remains the most effective peptide inhibitor of Grb7 developed to date.

## 1. Introduction

Cancer progression occurs with the dysregulation of critical signaling pathways controlling cell proliferation, migration, and survival. These often involve receptor and non-receptor tyrosine kinases that initiate and propagate these signaling cascades. Tyrosine kinases and their downstream interaction partners thus constitute targets for therapeutics development aimed to halt the aberrant behavior of the cancer cell [1,2]. In the case of breast cancer, HER2, that initiates signals for cell growth and survival, is already a major target of both antibody and tyrosine kinase inhibitor therapeutic intervention for HER2+ve cancers [3]. Interestingly, overexpression of the HER2 amplicon also results in the overexpression of the growth receptor bound protein 7 (Grb7) [4]. This adapter protein normally functions through interactions with FAK in the formation of focal adhesions that are important for cell migration [5,6]. When co-overexpressed with HER2, however, Grb7 is also found interacting directly with HER2 and activating pathways of cell proliferation and survival [7]. Grb7 overexpression has been determined as an independent indication of poor prognostic cancer progression [8,9,10,11]. Thus, the interaction of Grb7 with upstream receptor and non-receptor tyrosine kinases is a key strategic target for therapeutic intervention [12,13].

Grb7 is a dimeric 532 amino acid protein comprising an N-terminal proline rich (PR) domain, a Ras associating (RA) domain, a Pleckstrin homology (PH) domain and a C-terminal src homology (SH2) domain [14]. It is through the SH2 domain that Grb7 is able to detect the activation of upstream binding partners including HER2, FAK, SHC, and Eph1 [15]. Grb7 is able to recognize phosphotyrosines within a pYXN motif [12,16]. This is similar to the recognition sequence of the Grb2-SH2 domain that is the usual downstream adapter for HER2 [17], and thus Grb7 potentially outcompetes Grb2 for this site when overexpressed. The downstream interactions made by Grb7 are less well characterized and therefore it is the SH2 domain that has received attention for the development of inhibitory ligands [18]. These have usually been peptidic in nature, since peptides can confer higher specificity and affinity than small molecules due to their greater surface area coverage. In the case of SH2 domains, that all feature a highly positively charged pocket for the binding of the phosphotyrosine, it is unlikely that a small molecule would be able to confer specificity. Rather, a peptide molecule that binds across the whole recognition interface of the SH2 is required.

To this end, a series of peptides has been developed that target the SH2 domain of Grb7. The first specific peptide that was developed was a cyclic 11-residue peptide known as G7-18NATE [19]. This peptide was discovered with the use of phage display that selected for peptides in a turn conformation and with a YXN motif. It was subsequently synthesized as a monocyclic peptide with the use of a thioether linkage between its N-terminal end and the C-terminal cysteine sidechain. Interestingly, despite the tyrosine being unphosphorylated, G7-18NATE was found to be able to bind to the Grb7-SH2 domain with micromolar affinity [20]. Further investigation revealed that phosphate ions in solution could compensate for the lack of phosphate covalently attached to the tyrosine [21]. G7-18NATE showed specificity for the Grb7-SH2 domain over other closely related SH2 domains including Grb10, Grb14, and Grb2-SH2 domains and, indeed, over a whole array of other SH2 domains [21,22]. When synthesized with a cell penetrating peptide (CPP) tag attached (either Tat or Penetratin) [23], G7-18NATE was found to effectively inhibit cell proliferation, motility, cell invasion and 3D culture formation in several cancer cell lines [24,25,26] and reduce metastatic spread in an animal model of pancreatic cancer [27]. Interestingly, several of these effects were found to be synergistic with anticancer agents doxorubicin and trastuzumab [24,26]. Investigation of the disruption of cellular events showed that RAC1 and ERK pathways were inhibited by G7-18NATE [24,25]. Thus G7-18NATE was shown to be an effective inhibitor of Grb7 and a useful tool for the dissection of the molecular events occurring downstream.

**Figure 1:**
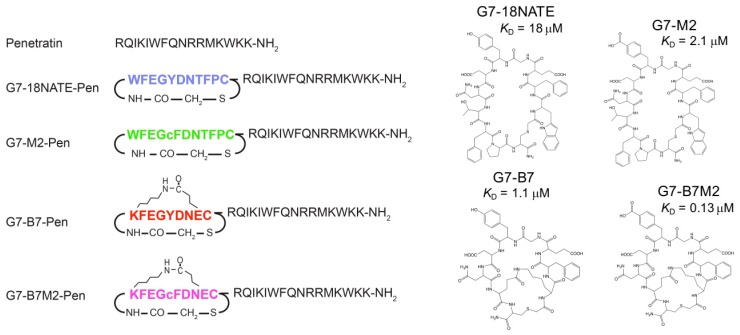
Schematic representation of peptides used in this study. G7-18NATE, G7-B7, G7-B7M2, and G7-M2 Pen-linked peptides are represented schematically alongside Pen on the LHS with single letter codes representing the amino acids and lactam and thioether linkages indicated with a chemical representation. The chemical structure of the peptides are shown on the RHS, along with the affinity of interaction (*K*_D_ in units of μM) of each of these peptides for the Grb7-src homology (SH2) domain previously determined in vitro. Naming guide: “G7” refers to Grb7 target peptides; “M2” refers to the incorporation of the carboxyphenylalanine (cmF) phosphotyrosine mimetic; “B7” refers to the seventh, and highest affinity, bicyclic peptide to be developed.

Subsequent G7-peptides were developed based on G7-18-NATE with improved affinity and stability in solution (Figure 1 and Supporting Information Table S1). Such improvements were anticipated to make the G7-peptide even more useful for cellular studies and potentially viable for therapeutic use. Structural studies of G7-18NATE in complex with the Grb7-SH2 domain revealed the conformational arrangement adopted in its bound state [28]. This led to a series of changes made to the G7-peptide. Firstly, phosphotyrosine mimetics in the forms of carboxymethylphenylalanine (M1) and carboxyphenylalane (M2) led to improved binding affinity of peptides G7-M1 (*K*_D_ = 5.7 μM) and G7-M2 (*K*_D_ = 2.1 μM) for the Grb7-SH2 domain compared to G7-18NATE (*K*_D_ = 18.1 μM) [29]. Secondly, a linkage tethering amino acids 1 and 8 was added to confer rigidity to the G7-peptide without removing amino acid sidechains required at the protein interface [30]. The first of these contained a staple group that conferred higher affinity, but also changed the mode of interaction with the Grb7-SH2 domain target [31]. This led to a further constraint of the peptide by the removal of two amino acids leading to a series of bicyclic peptides with even higher binding affinities [32]. The highest affinity bicyclic peptide, G7-B7 (*K*_D_ = 1.1 μM), contained a lactam linkage and, when synthesized in combination with the M2 mimetic, resulted in G7-B7M2 with binding affinity in the nanomolar range (*K*_D_ = 0.13 μM) representing an overall 140-fold improvement in binding affinity compared to G7-18NATE. The G7-peptides were all also confirmed to retain their specificity for Grb7-SH2 domain over other closely related SH2 domains, and thus represent the highest affinity specific ligands against Grb7 developed to date.

Penetratin (henceforth referred to as “Pen”)-linked versions of these G7-peptides were also prepared, and shown to enter cells and inhibit the ability of Grb7 to interact with upstream binding partners HER2, FAK, and SHC [32]. The current work, therefore, was undertaken in order to determine the ability of the G7-peptides to exert biological effects in cancer cells. Two cell lines were selected, the HER2+ve cell line SKBR-3 that massively overexpresses Grb7 along with HER2, and MDA-MB-231 that is a triple negative breast cancer cell (TNBC) line that expresses only low levels of Grb7. Both cell lines have previously been investigated for the effect of Grb7 knockdown [33]. Reductions in cell growth, migration and invasion were observed for both cell lines, indicating the potential of G7-Pen peptides to combat the aberrant behavior of both HER2+ve and TNBC cancer cells. Here, we were interested to determine whether the enhanced binding affinity of the most recently developed G7-peptides would result in greater potency in cellular assays of cell proliferation, migration and invasion compared to G7-18NATE-Pen. Surprisingly, our study revealed activity for the earlier generation monocyclic G7-peptides but relatively little activity for the higher affinity bicyclic G7-peptides. We first considered whether this was due to steric hindrance by the Pen sequence in the case of the bicyclic peptides. Binding studies using isothermal titration calorimetry (ITC) showed that this was not the case. We then investigated whether the differences in activity were likely due to differences in the ability of the peptides to interact with cellular membranes and enter the cell. For this we used surface plasmon resonance (SPR) to examine binding to lipid bilayers and mass spectrometry to quantitate cell uptake. We discovered that greater activity of G7-18NATE-Pen correlated with its superior cellular permeability over the bicyclic peptides, highlighting that even a small change to a cargo can affect its successful delivery by CPPs and thus its effective activity.

## 2. Results

### 2.1. Preparation of G7-Peptides

The G7-peptides selected for the current study included the first generation G7-18NATE peptide as a benchmark Grb7 inhibitor, and three modified peptides that have previously been shown to have higher affinity for the Grb7-SH2 domain in vitro. These were the G7-M2, G7-B7, and G7-B7M2 peptides (Figure 1). The G7-M2 peptide is identical to G7-18NATE except for the inclusion of a phosphotyrosine mimetic carboxyphenylalanine (cF) in place of tyrosine at position 5. The G7-B7 peptide is bicyclic, constrained via a lactam built between residues 1 and 8 and shorter than G7-18NATE by 2 amino acids. The G7-B7M2 peptide combines both of these modifications, overall enhancing the in vitro binding affinity for the Grb7-SH2 domain 140 times [32]. Each peptide was prepared both separately and in tandem with the CPP Pen positioned C-terminal to the amino acid sequence. In addition, Pen alone was also prepared as a control. Peptides were synthesized using solid phase peptide synthesis, with lactam formation conducted on-resin and thioether formation performed after cleavage from the resin, as previously reported [32]. All peptides were purified to greater than 95% purity using rpHPLC and verified using mass spectrometry. Further verification of the structure of the separate peptides was provided by crystal structure analyses of these peptides in complex with the Grb7-SH2 domain [28,29,32].

### 2.2. Effect of G7-Peptides on Signaling

**Figure 2:**
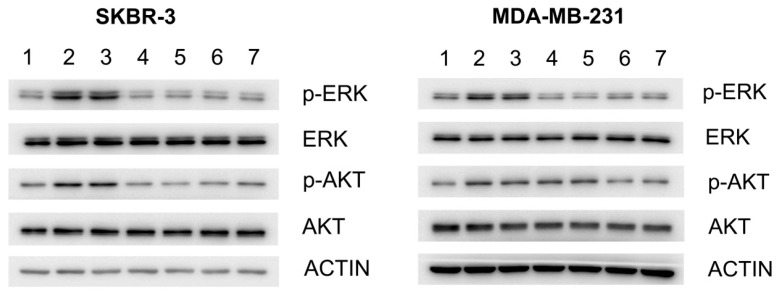
Effect of G7-peptides on ERK and AKT Signaling in (**left**) SKBR-3 and (**right**) MDA-MB-231 cell lines. (lane 1, non-stimulated; lane 2, FN; lane 3, FN+Pen; lane 4, FN+G7-B7-Pen; lane 5, FN+G7-B7M2-Pen; lane 6, FN+G7-M2-Pen; lane7, FN+G7-18NATE-Pen). β-Actin was used as a loading control. The results shown are representative of three independent experiments. FN = fibronectin.

In order to determine whether the G7-peptides could impact Grb7 mediated signaling pathways, we examined their effect on downstream ERK and AKT activation in fibronectin stimulated SKBR-3 and MDA-MB-231 breast cancer cell lines. Cells were treated with 20 μM peptide and phosphorylation of signaling molecules assessed by Western blot using antibodies against both non-phosphorylated and phosphorylated versions of the peptides (Figure 2). This showed that while fibronectin stimulated cells contained increased levels of p-ERK and pAKT (compared to non-stimulated control) and so did cells treated with the Pen only negative control, most of the G7-peptide treated cells did not (Figure 2). In SKBR-3 cells there was clear inhibition of ERK and AKT phosphorylation by all four peptides. In MDA-MB-231 cells, the same effect could be seen, except for inhibition of AKT phosphorylation by the bicyclic peptides (G7-B7-Pen and G7-B7M2-Pen). Interestingly, basal levels of pERK and pAKT were not affected. Thus, altogether, all four G7-peptides appeared to have the ability to enter cells and to inhibit the fibronectin stimulated activation of ERK and/or AKT. In these experiments, however, there was no trending difference between the inhibitory effects of the four different G7-peptides. This was unexpected, since the peptides possess a 100-fold range of affinity for the Grb7 target in vitro (from *K*_D_ = 18 μM to 0.13 μM) and were therefore anticipated to have differential effects on signaling.

### 2.3. Effect of G7-Peptides on Cell Proliferation

**Figure 3:**
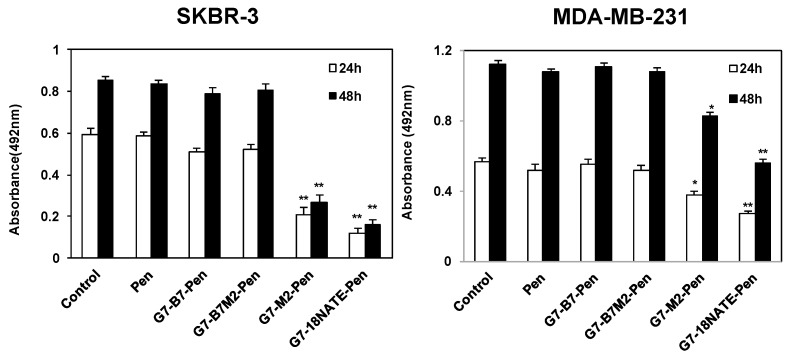
Effect of G7-peptides on proliferation of (**left**) SKBR-3 and (**right**) MDA-MB-231 cells. Cells were treated with 20 μM control peptide (Pen) or 20 μM G7-peptide inhibitors (G7-B7-Pen, G7-B7M2-Pen G7-M2-Pen and G7-18NATE-Pen) for 24 h and 48 h at 37 °C. Cell proliferation was examined with the MTS assay. Values are means ± SEM for at least three independent experiments with duplicate samples. A student′s t test was performed between control (no peptide) and G7-peptide treated samples with * *p* < 0.05, ** *p* < 0.01.

We then undertook to determine the degree to which the G7-peptides were able to inhibit cell proliferation. The SKBR-3 and MDA-MB-231 cell lines were treated with 20 μM of each peptide and cell proliferation assessed after 24 and 48 h. In this case, we observed a potent ability of the G7-18NATE-Pen and the G7-M2-Pen to inhibit cell proliferation in SKBR-3 cells (and a lesser but significant inhibition in MDA-MB-231 cells), but, surprisingly, no evidence of inhibition by the bicyclic inhibitors G7-B7-Pen and G7-B7M2-Pen (Figure 3). Treatment by these peptides resulted in the same degree of cell proliferation of cells as untreated cells and the Pen only treated control.

### 2.4. Effect of G7-Peptides on Cell Migration

**Figure 4:**
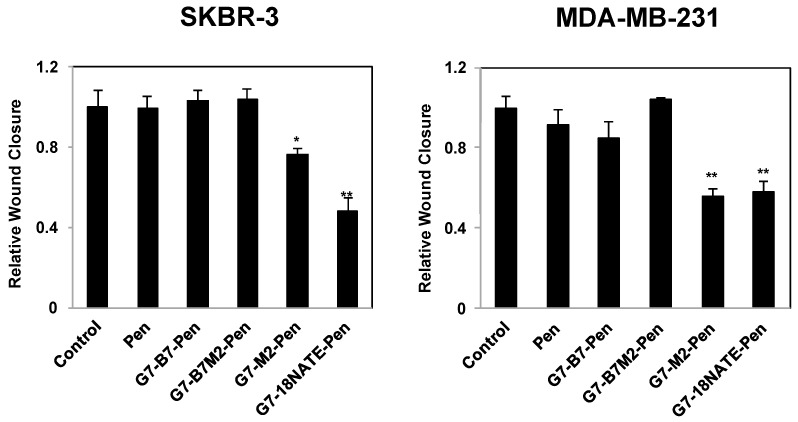
Effect of the G7-peptide inhibitors on (**left**) SKBR-3 and (**right**) MDA-MB-231 cell migration using wound healing assay. SKBR-3 and MDA-MB-231 cells were treated with 20 μM of the control peptide (Pen) or 20 μM G7-peptide inhibitors (G7-B7-Pen, G7-B7M2-Pen G7-M2-Pen and G7-18NATE-Pen). Cell migration was analyzed using the wound-healing assay, in which a scratch wound was introduced into a confluent monolayer of SKBR-3 or MDA-MB-231 cell lines and the extent of wound closure monitored after 48 h (SKBR-3) or 8 h (MDA-MB-231). Relative wound closure is expressed relative to the untreated control MDA-MB-231 and SKBR-3 cells, which is normalized to 1.0. Bars represent means ± SEM for at least three independent experiments with duplicates. A student’s t-test was performed between control (no peptide) and G7-peptide treated samples with * *p* < 0.05, ** *p* < 0.01.

**Figure 5:**
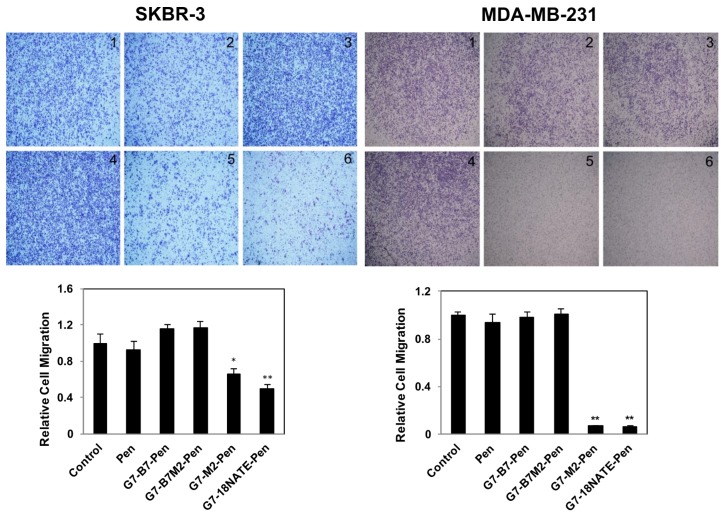
Effect of the G7-peptide inhibitors on MDA-MB-231 and SKBR-3 cell migration using a Transwell assay. SKBR-3 and MDA-MB-231 cell lines were treated with 20 μM of the control peptide (Pen) or 20 μM G7-peptide inhibitors for 30 h (SKBR-3) or 4 h (MDA-MB-231) at 37 °C. Cell motility was measured using the Transwell assay. Top: Representative images of migrated SKBR-3 and MDA-MB-231 cells (image 1, Control; 2, Pen; 3, G7-B7-Pen; 4, G7-B7M2-Pen; 5, G7-M2-Pen; 6, G7-18NATE-Pen). Bottom: Migrated cells are expressed relative to the untreated control MDA-MB-231 and SKBR-3 cells, which is normalized to 1.0. Bars represent mean ± SEM for at least three independent experiments with duplicates. A student′s t-test was performed between control (non-treated) and G7-peptide treated samples with * *p* < 0.05, ** *p* < 0.01.

The G7-peptides were next tested for their ability to inhibit cell migration, as has previously been shown to occur upon Grb7 knockdown in SKBR-3 and MDA-MB-231 cell lines [33]. Cells were treated with G7-peptide or control peptide Pen at 20 μM concentration. Again, while G7-18NATE-Pen and G7-M2-Pen peptides were found to reduce cell migration as assessed by the wound healing assay (Figure 4) and the Transwell Motility Assay (Figure 5), the bicyclic peptides G7-B7-Pen and G7-B7M2-Pen did not. We observed a seeming trend of enhanced cell motility in the SKBR-3 line, but this enhancement was not statistically significant. Wound closure by G7-18NATE-Pen and G7-M2-Pen peptides was reduced by about 50% in both cell lines, which is similar to the effect of Grb7 knockdown [33]. Transwell migration, which additionally assesses the ability of the cells to migrate towards a chemoattractant, showed that only the G7-18NATE-Pen and G7-M2-Pen peptides were able to significantly decrease the ability of the cells to migrate towards FBS. The effect appeared to be more potent in MDA-MB-231 cells than in SKBR-3 cells.

### 2.5. Effect of G7-Peptides on Invasion

**Figure 6:**
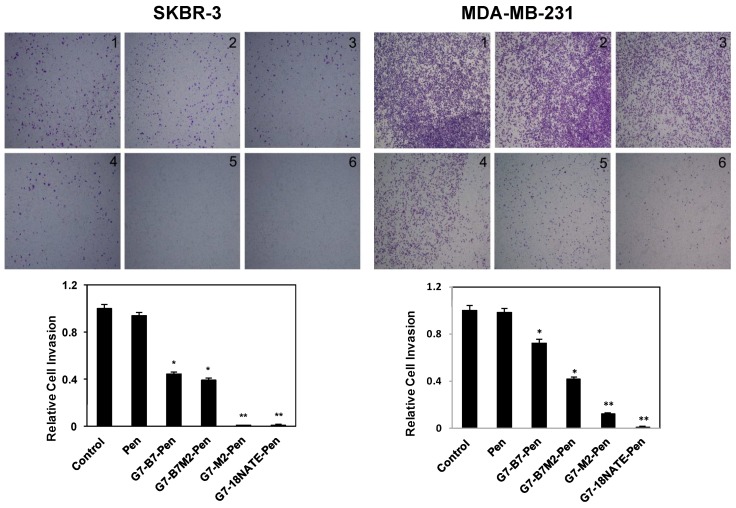
Effect of the Grb7 peptide inhibitors on (**left**) SKBR-3 and (**right**) MDA-MB-231 cell invasion. Top: Representative images of the Transwell invasion assay demonstrating that 20 μM G7-peptide inhibitors for 48 h (SKBR-3) or 24 h (MDA-MB-231) inhibit invasion through the Matrigel-coated filters (image 1, Control; 2, Pen; 3, G7-B7-Pen; 4, G7-B7M2-Pen; 5, G7-M2-Pen; 6, G7-18NATE-Pen). Bottom: Relative cell invasion is expressed relative to the untreated control cells, which is normalized to 1.0. Bars represent mean ± SEM for at least three independent experiments with duplicate samples. A student′s t test was performed between control and G7-peptide treated samples with * *p* < 0.05, ** *p* < 0.01.

Finally, the peptides were also tested for their ability to inhibit cell invasion in both experimental cell lines (Figure 6). In addition to migration this assay tests the ability of the cells to penetrate a layer of extracellular matrix protein. SKBR-3 cells and MDA-MB-231 cells were treated with the G7-peptides at 20 μM concentration and their ability to move through the Matrigel-coated filters determined after 48 h. In this case highly potent activity was observed for the G7-18NATE-Pen and G7-B7M2-Pen peptides, with greatly reduced ability of the cells to invade, and some inhibitory activity was also observed for the G7-B7-Pen and G7-B7M2-Pen peptides in both cell lines. No activity was observed upon treatment by the Pen peptide control.

Altogether, the cellular assays comparing the potency of the G7-peptides revealed that while the G7-18NATE-Pen and G7-M2-Pen peptides were able to inhibit cell proliferation, migration and invasion in both cell lines, the bicyclic peptides G7-B7-Pen and G7-B7M2-Pen showed no, or significantly reduced, potency. This was unexpected since, as previously explained, the bicyclic peptides have been shown to possesses higher binding affinity to the Grb7-SH2 domain in in vitro assays than the first generation monocyclic G7-18NATE-Pen and G7-M2-Pen peptides. We therefore commenced to determine the basis for this seemingly paradoxical outcome.

### 2.6. Potential Differential Effect of the Penetratin Cell Permeability Tag

We considered the fact that the in vitro testing of the ability of the G7-peptides to bind to the Grb7-SH2 domain was previously conducted for the G7-peptides in the absence of the Pen cell permeability sequence. It was possible that, due to the different binding mode of the bicyclic G7-peptides to the Grb7-SH2 domain compared to the monocyclic peptides, the Pen may act to sterically hinder the Grb7-SH2 interactions in the case of the bicyclic peptides. In particular, the X-ray crystallographic analysis of the G7-B7 peptide in complex with the Grb7-SH2 domain identified that the C-terminal cysteine in the G7-B7 peptide is positioned closer to the protein surface than the C-terminal cysteine in G7-18NATE and that the C-terminal amide group (present due to the synthetic procedure) contributed to Grb7-SH2 domain interactions that were not observed for G7-18NATE [28,32]. Thus it was possible that the presence of the Pen sequence, that is synthesized at the C-terminal end of the G7-peptide construct, could differentially interfere with target binding.

To investigate this possibility, the binding of the Pen coupled peptides to Grb7-SH2 was measured using isothermal titration calorimetry (ITC). Initial experiments were trialed by surface plasmon resonance (SPR), however, severe non-specific binding effects were observed between the positively charged Pen and the negatively charged CM5 chip (results not shown). The ITC experiment was conducted as previously described [30]. The measurements were made for just G7-18NATE and G7-B7 as representatives of the monocyclic and bicyclic G7-peptides.

**Figure 7:**
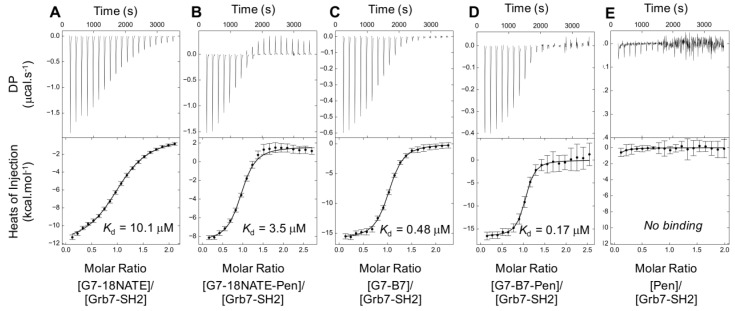
Isothermal titration calorimetry (ITC) analysis of Grb7-SH2 and G7-targeting peptides. Thermograms of (**A**) G7-18NATE, (**B**) G7-18NATE-Pen, (**C**) G7-B7, (**D**) G7-B7-Pen, (**E**) Pen titrated into the Grb7-SH2 domain solution in a buffer comprising 1 mM Na_3_PO_4_, 20 mM HEPES, 150 mM NaCl, 4 mM β-mercaptoethanol (pH 7.4). The bottom panel comprises the data after integration of the peaks and, if necessary, subtraction of the heat of dilution control (peptide into buffer titration). The binding curve shows the fit to a single-site binding model and the *K*_D_ of interaction corresponding to this fit in units of μM. Error bars shown display the uncertainty in baseline calculations. DP = differential power. Note: Although an air bubble occurred half way through the injection series, it was clear that Pen does not bind on its own (or binds at very low affinity) to the Grb7-SH2, with minimal heat generated upon titration of Pen.

The interactions between Grb7-SH2 and G7-peptides were found to be exothermic with a release of heat following each titration (Figure 7). The curve analysis allowed the affinity of interactions to be determined for all peptides (Table 1). Binding to Grb7-SH2 by G7-18NATE and G7-B7 was determined to be *K*_D_ = 10.1 μM and *K*_D_ = 0.48 μM respectively, similar to values previously reported [29,32]. Binding by the Pen-linked peptides was determined to be *K*_D_ = 3.45 μM and *K*_D_ = 0.17 μM for G7-18NATE-Pen and G7-B7-Pen respectively, showing that, in both cases, the inclusion of the Pen sequence did not detract from binding affinity. In fact, it resulted in an approximately two-fold increase in binding affinity to Grb7-SH2 (although binding by the Pen peptide alone could not be detected) (Figure 7E).

This experiment demonstrated that the addition of the Pen CPP to the G7-bicyclic peptide did not negatively impact its ability to bind to the Grb7-SH2 domain, and thus was unlikely to be the reason for the reduced cellular activity observed.

### 2.7. Investigation of G7-Peptide Cellular Uptake

Next it was considered whether different levels of cell permeability could underlie the differential activities of the monocyclic vs. bicyclic peptides. Even though Pen has been used widely as a CPP for the transport of peptide cargos across cell membranes and into the cytosol of the cell, the efficiency of cell penetration has been observed to be affected by the nature of the cargo [34,35]. Although we previously observed the ability of all of the G7-peptides to block Grb7 interactions with upstream binding partners in SKBR-3 cells [32] and here showed the ability of all four peptides to impact cellular signaling, it was possible that, under the conditions utilized for cellular assays of cell proliferation, migration, and invasion, the cell penetration of the G7-bicyclic peptides was a limiting factor.

We therefore measured the ability of the G7-peptides to interact with model membranes and be delivered into cells. Previous studies of cellular uptake by peptides, including our own, have utilized fluorescent labelling to visualize and measure cell uptake [36,37]. These methods can, however, be misleading for several reasons. Firstly, the inclusion of the fluorescent label can alter the overall hydrophobicity, hydrophilicity, and charge of the construct to influence its uptake and even cytosolic localization [38,39]. Secondly, any protease activity in the cell culture medium or inside the cell can result in the separation of the fluorescent tag from the full-peptide, resulting in the fluorescent signal not being indicative of the peptide location. We therefore elected to use methods that did not involve any chemical modification of the G7-peptide constructs.

**Figure 8:**
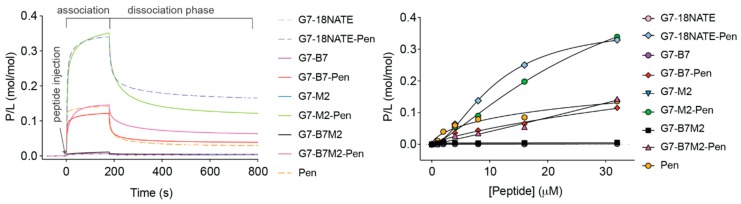
Binding of G7-peptides with model membranes as studied using surface plasmon resonance. Lipid bilayers composed of POPC/POPS (4:1 molar ratio) were deposited onto an L1 chip and peptide samples were injected for 180 s (association phase) and their dissociation followed for 600 s (dissociation phase). Response units (RU) signal obtained from surface plasmon resonance (SPR) were converted into moles of peptide and normalized to the lipid deposited onto the chip surface (1 RU = 1 pg/mm^2^) to obtained peptide-to-lipid-ratios (P/L). Left panel shows sensorgrams obtained upon injection of 32 μM peptide samples. Right panel shows dose-response curves in which the P/L obtained at the end of association phase (t = 170 s) is plotted as a function of peptide sample concentration injected over the lipid bilayer.

The mechanism used by Pen constructs to enter cells is dependent on the conditions of the assay (e.g., concentration, cargo, cell type), which can favor direct translocation across the membrane or entry via endocytic-mediated processes [40]. Either way, reaching the cytosol depends upon the ability of the peptide to interact with lipid bilayers, whether the cell membrane or that of endosomes. Thus, to examine whether ability to bind to lipid membranes might play a role in the effective activity of the G7-peptides, we compared the degree to which the G7-bicyclic and G7-monocyclic peptides interacted with model lipid membranes using SPR. This has been shown previously to be highly indicative of the ability of peptides to cross cellular membranes [41,42,43,44]. We compared the affinity of all four G7-peptides, both in their Pen linked and non-Pen linked forms and at concentrations ranging from 1 to 32 μM peptide concentrations, with negatively-charged membranes composed of POPC/POPS (4:1 molar ratio) (Figure 8). POPC (1-palmitoyl-2-oleoyl-sn-glycero-3-phosphocholine) is the most common phospholipid in the outer leaflet of the mammalian plasma membrane [45,46] and mimics the overall fluidity and neutral surface of healthy eukaryotic cells [47]. Phospholipids containing negatively-charged PS-headgroups, such as POPS, are normally restricted to the inner leaflet in eukaryotic cell membranes but become exposed at the cell surface of cancerous cells [48,49]. Thus, these model membranes were used to mimic the overall fluidity and the negative charge of cancer cell membrane surfaces.

This revealed three types of responses: firstly, no interaction with the lipid bilayer was observed for any of the G7-peptides that were non Pen-linked (G7-M2, G7-B7M2, G7-B7, G7-18NATE). This indicated that none of the peptides are able to interact with these model membranes without being linked to Pen. There was an intermediate binding interaction observed for Pen, G7-B7M2-Pen and G7-B7-Pen, suggesting that when the bicyclic G7-peptides are linked to Pen they have a similar affinity for lipid membranes as Pen alone. The most striking response, however, was the distinctly stronger binding interaction observed for G7-18NATE-Pen and G7-M2-Pen. These monocyclic peptides, when linked to Pen, were found to have an apparent enhanced ability to interact with membranes. These SPR experiments thus revealed a striking difference between the ability of the G7-bicyclic and G7-monocyclic Pen-linked peptides to interact with the model membranes. This is likely to also reflect a differential ability of the peptides to enter cells.

To directly compare the ability of the G7-peptides to enter cells, we used a mass spectrometric approach to detect and quantify peptide uptake. Here, HeLa cells were selected owing to their prevalent use as a model cell line both for cancer and for examining cellular uptake of peptides [50]. The cells were incubated with 20 μM peptide for 1 h at 37 °C, and mass spectrometry was used to detect and quantify peptide uptake. For this experiment G7-B7M2-Pen was compared with G7-18NATE-Pen and G7-18NATE alone. Briefly, following incubation, a sample of the buffer was reserved and the cells were rinsed of the remaining non-internalized peptide. Peptide remaining in the buffer solution, as well as total cell associated peptide, supernatant associated peptide (i.e., in the cytosol or in endosomes) and pelleted peptide (i.e., peptide associated with membranes) were quantified via mass spectrometry using previously established methods [41]. Quantitative comparison of G7-18NATE, G7-18NATE-Pen, and G7-B7M2-Pen distribution are shown in Table 2 and Table 3, as well as Supplementary Table S2 and Figure S2.

Table 2 shows the concentration of peptide associated with the HeLa cell supernatant or pellet compared to the total peptide detected. G7-18NATE was not detected in any of the cellular fractions suggesting that G7-18NATE does not internalize inside cells, nor associate with the cell surface. G7-18NATE-Pen however was detected at approximately 200 and 300 nM concentrations in the supernatant and pelleted fractions respectively, suggesting that it distributes between a soluble form (in the cytosol or in organelles) and a membrane-associated form. G7-B7M2-Pen was not detected in the supernatant fraction, but appeared at approximately 100 nM in the pelleted fraction. This is consistent with G7-B7M2-Pen having a poorer ability to interact with membranes compared with G7-18NATE-Pen.

Table 3 summarizes these same data in terms of the percentage of total unbound G7-peptide (11 μM measurable in buffer at the end of the 1 h incubation) associated with the supernatant, cell pellet or total cell. This equates to 6.2% of the G7-18NATE-Pen and 1.5% of G7-B7M2-Pen being associated with the cells. Interestingly, these values are both higher than the approximately 1% volume occupied by the HeLa cells in the peptide incubation solution. This therefore indicates that peptide sequestered from the surrounding solution tends to accumulate in the cell, rather than reaching an equilibrium throughout the cell and solution volume. Furthermore, it shows that G7-18NATE-Pen is highly efficient w.r.t. cell uptake, and that G7-B7M2-Pen is less so.

Overall these results demonstrate that the G7-peptides possess distinct abilities in terms of their interactions with mammalian cell membranes and in penetrating cells. The mono-cyclic G7-peptides (G7-18NATE-Pen and G7-M2-Pen) are clearly superior to the bicyclic peptides (G7-B7-Pen and/or G7-B7M2-Pen) in both these regards. This is, therefore, likely to be the reason for the greater activity observed for the mono-cyclic G7-peptides in SKBR-3 and MDA-MD-231 cells over the bicyclic G7- peptides.

## 3. Discussion

Grb7 is an attractive target for the development of novel anti-cancer agents that may act on their own or in combination with other therapies. First generation peptide inhibitors have been developed that target the Grb7-SH2 domain by mimicking upstream phosphotyrosine binding partners. The G7-18NATE peptide, in particular, has provided significant proof-of-concept evidence that inhibition of Grb7 via blocking its SH2 domain has effective anti-cancer effects in cells and animal models [24,25,26,27]. A high affinity inhibitor (with a *K*_D_ in the nanomolar range or lower) is required, however, to facilitate further testing or have therapeutic potential. A series of higher affinity Grb7-targeted peptides, known as G7-peptides, has therefore been developed. In particular, second generation peptides including G7-M2 (with a carboxyphenylalanine (cF) phosphotyrosine mimic), G7-B7 (a bicyclic G7-peptide tethered to form the bound conformation) and G7-B7M2 (a bicyclic G7-peptide that also incorporates cF) have been shown to possess increasingly improved binding affinities (from 9 to 140-fold higher) for Grb7-SH2 over G7-18NATE.

The current study was therefore initiated to test whether the higher affinity G7-peptides are, in fact, more potent in cellular assays than the original G7-18NATE peptide. In order to measure cellular activities, the G7-peptides were prepared with the covalently attached CPP Pen. It is the most frequently used CPP in previous studies of G7-18NATE activity, and was therefore also selected for the current study [24,25,26,27]. We had previously shown that G7-peptides were able to disrupt Grb7 interactions with upstream binding partners, but it was not yet known whether the second generation peptides possess improved anti-cancer activity over G7-18NATE-Pen [32].

We began by applying the peptides to SKBR-3 (HER2+ve breast cancer cell line) and MDA-MB-231 (TNBC cell line) and examined the phosphorylation status of signaling molecules downstream of Grb7. We observed that the G7-peptides all inhibited the fibronectin stimulated phosphorylation of ERK and AKT in SKBR-3 cells. A similar, but less striking, observation was made in MDA-MB-231 cells. It is interesting that the peptides were effective in both the SKBR-3 cell line, that massively overexpresses Grb7, as well as the MDA-MB-231 cell line, in which relatively low levels of Grb7 are expressed. It suggests that the potency of the peptides is sufficient to have an effect even when Grb7 is present at relatively high concentrations. The results are consistent with the lowered ERK activation seen in MDA-MB-468 cells upon G7-18NATE-Pen treatment [25] and lowered activation of STAT3, AKT, ERK, JNK and p38 in SKBR-3 cells upon Grb7 knockdown [51]. What was unexpected, however, was that the second generation peptides (G7-M2-Pen, G7-B7-Pen and G7-B7M2-Pen) were not more active than the first generation G7-18NATE-Pen, despite their greater binding affinities for the Grb7-SH2 domain measured in vitro.

We then examined the effect of the G7-peptides on cell proliferation, cell migration and cell invasion. Again, all studies were conducted using both SKBR-3 and MDA-MB-231 cell lines. We consistently observed effective inhibition by the G7-18NATE-Pen peptide, similar to the effect of Grb7 knockdown and similar to other studies of the activity of G7-18NATE [24,25,26,33]. The G7-M2-Pen peptide showed a similar inhibitory effect in these assays, despite its 9-fold increased binding affinity (*K*_D_ = 2.1 μM) for the Grb7-SH2 domain over that of G7-18NATE (*K*_D_ = 18 μM). The lack of enhanced activity of G7-M2-Pen suggested that either the cellular responses being measured were not sensitive to the differential affinity of the peptides, that the effective in vivo affinity of G7-M2-Pen was not enhanced over G7-18NATE-Pen (potentially due to the presence of cellular phosphate that can substitute for a phosphomimetic), or that poorer cellular uptake of the more highly charged peptide countered its enhanced affinity for the target.

More surprising, however, was the lower activity determined for the bicyclic G7-peptides, G7-B7-Pen and G7-B7M2-Pen. Since these peptides have been shown to have 18-fold and 140-fold higher affinity respectively for the Grb7-SH2 target in vitro, it was expected that they should have enhanced activity over G7-18NATE-Pen. Instead, no significant inhibition of cellular proliferation or migration in SKBR-3 or MDA-MB-231 cells was observed. In the case of the cellular invasion assays, some inhibition was achieved by the bicyclic G7-B7-Pen and G7-B7M2-Pen peptides, but inhibition was less than that achieved by the monocyclic G7-18NATE-Pen and G7-M2-Pen peptides.

This unexpected outcome prompted us to consider whether there was an inadvertent detrimental effect of the covalently attached Pen sequence on the binding of the bicyclic peptides to the Grb7-SH2 domain target. Previous binding assays had been conducted for the cyclic peptides in the absence of the CPP. X-ray crystallographic structural studies of G7-B7 and G7-B7M2 peptides in complex with the Grb7-SH2 domain show that the C-terminal cysteine of these bicyclic peptides is close to the binding surface of the protein, whereas the C-terminal cysteine of G7-18NATE is positioned away from the protein surface [28,32]. It was therefore possible that the presence of the CPP that is attached at this point could interfere with peptide binding in the case of the bicyclic peptides. Binding studies using ITC showed that this was not the case, with covalently attached Pen not adversely affecting the binding affinity of either G7-18NATE or G7-B7. In fact, binding affinity was slightly enhanced in both cases suggesting an additional interaction conferred by Pen (though no binding could be detected for Pen alone).

We therefore considered that the lower activities of the bicyclic peptides may be due to their poorer ability to be taken up into cells. Despite having observed the ability of the bicyclic peptides to block Grb7 interactions with binding partners [32] and, in this study, to inhibit downstream phosphorylation events, there was no evidence of enhanced activity by these higher affinity peptides. It was possible that the cellular conditions utilized to study signaling, such as induced by overnight serum starvation, were more permissive to uptake of peptide than the conditions utilized for the activity studies. Indeed, previous investigations have detected that serum starvation can sensitize cells to peptide uptake [52]. The differences in peptide ability to inhibit cellular proliferation, migration and invasion may have reflected difference in cellular uptake under these more stringent conditions. Thus, peptide interactions with model membranes composed of POPC/POPS (4:1) were investigated using SPR. The ability of the peptides to interact with membranes was in remarkable correlation with the peptide activities observed, with the highest sensorgram responses observed for the monocyclic peptides G7-18NATE-Pen and G7-M2-Pen. Moderate interactions were observed for the bicyclic peptides G7-B7-Pen and G7-M7M2-Pen as well as Pen alone (and no interactions were observed for the peptides lacking a Pen sequence, as expected). Since the entry of peptides into cells involves crossing a lipid bilayer, the ability of a peptide to interact with a lipid bilayers can be considered a good indication of this ability. Furthermore, the negatively-charged membranes composed of POPC/POPS (4:1) mimic the overall fluidity and the negatively-charged surface of cancer cells.

This was further supported by direct mass spectrometric detection of G7-18NATE-Pen vs. G7-B7M2-Pen uptake into HeLa cells, demonstrating their association with supernatant or membranes of the cells. G7-18NATE-Pen showed higher association with the total content of HeLa cells compared to G7-B7M2-Pen, and also detectable amounts in the supernatant of the cells (i.e., in soluble form). This is consistent with the G7-18NATE-Pen peptide not only interacting with membrane, but also being able to traverse it into the cytosol of the cell. The G7-B7M2-Pen peptide, in contrast, was not detected in the supernatant fraction of the HeLa cells. The percentage of available peptide associated with the cells was 6.2% for G7-18NATE and 1.5% for G7-B7M2-Pen. Interestingly, this is greater than the approximated percentage volume occupied by the cells in the experiment, reflecting the one-way uptake of the peptide into cells.

Together these experiments show a superior ability of the monocyclic peptides to interact with lipid bilayers and enter cells, compared to the bicyclic peptides. Lack of cellular uptake would explain the loss of, or limited activity of, the bicyclic peptides in cell proliferation, migration and invasion assays. The reason for the poor cell-permeability of the bicyclic peptides is not yet known. It may be that the intrinsically constrained structure of the bicyclic peptides interferes with membrane interactions. It is the case, however, that other bicyclic constrained peptides have successfully traversed cellular membranes to exert their intracellular activity [34,53] and some bicyclic peptides have been designed to enhance cell-permeability [54]. Thus, if peptide constraint interferes with the G7-bicyclic peptide cell permeability, it is particular to this class of peptide. Alternatively, it may be that changes to the overall peptide cargo lipophilicity affect its ability to enter cells. In the course of development of the G7-bicyclic peptides, two amino acids (Trp1 and Thr8) were replaced by Lys and Glu respectively to form a lactam ring and two amino acid residues (Phe9 and Pro10) were removed to further constrain the peptide [31]. It may be that these residues contributed to productive membrane interactions. In particular, it is known that aromatic residues serve an important role in membrane insertion [55]. Thus, in the process of enhancing the binding affinity of the developed bicyclic G7-peptides, their cell permeability has been adversely affected. Their application as Grb7 inhibitors therefore may depend upon an alternative strategy for cellular delivery, potentially through the use of CPPs with ability to deliver cargos into the cytosol more efficiently than Pen [56,57]. Indeed, previous work in our laboratory has suggested that the addition of a nuclear localization sequence (that is highly lysine and arginine rich) dramatically increases the cellular uptake of penetratin [37] and could be used for delivery of the bicyclic G7-peptides.

In summary, these studies highlight the considerations that must be made in the development of peptide inhibitors to intracellular targets and demonstrate that G7-18NATE-Pen remains the most effective peptide inhibitor of Grb7 developed to date.

## 4. Materials and Methods

### 4.1. Cell Line and Reagents

The human breast cancer cell line SKBR-3 was obtained from the American Type Culture Collection (ATCC, Manassas, VA, USA); the MDA-MB-231 cell line was obtained from EG & G Mason Research Institute (Worcester, MA, USA). Both cell lines were maintained according to ATCC guidelines. Antibodies against rabbit phospho-Ser473 AKT (catalog No.4058), rabbit AKT (catalog No. 4685), rabbit phospho-Thr202/Tyr204 ERK (catalog No. 4370) and rabbit p44/42MAPK(ERK1/2) (catalog No. 4695) were purchased from Cell Signaling Technology (Beverly, MA, USA). Antibodies against β-actin (catalog No. sc-69879) were purchased from Santa Cruz Biotechnology (Dallas, TX, USA). Donkey anti-mouse HRP, and goat anti-rabbit HRP were obtained from Bio-Rad Abcam (Hercules, CA, USA).

### 4.2. Western Blotting

Cells were serum starved overnight and treated for 1 h with G7-targeting peptides (20 μM) prior to stimulation for 10 min with fibronectin (10 μg/mL). Cells were washed twice with ice-cold PBS and then lysed with modified RIPA buffer (50 mM Tris-HCl pH 8.0, 150 mM NaCl, 1% (*v/v*) NP40, 0.1% (*w/v*) SDS, 0.5% (*w/v*) deoxycholate, 10% (*v/v*) glycerol, 5 mM EDTA, 20 mM NaF and freshly added aprotinin (10 mg/mL), 1 mM PMSF, leupeptin (10 mg/mL), 1 mM sodium orthovanadate). The total protein concentration was determined using the BradfordUltra reagent from Expedeon (catalog No. BFU1L, Over, Cambridge, UK). Protein samples were subjected to SDS-PAGE (8–12%) gel according to the molecular size of target protein, and electrophoresis and membrane transfer was performed following the manufacturer′s protocol (Bio-Rad, Hercules, CA, USA). The primary antibodies were incubated overnight at 4 °C in TBS-T (10 mM Tris pH 7.5, 150 mM NaCl, 0.05% (*v/v*) Tween-20), and the corresponding secondary antibodies were incubated for 1 h at RT in TBS-T, with three washes after each incubation. ECL reagents were used to show the positive bands on the membrane.

### 4.3. Cell Proliferation Assay

MDA-MB-231 (5 × 10^3^ cells/well) or SKBR-3 (1 × 10^4^ cells/well) cells were seeded in 96-well plates and the Cell Titer (96 Aqueous One solution cell proliferation assay (MTS assay); Promega Corp. Madison, WI, USA, Cat No. G3580) was used to evaluate cell proliferation. Assays were performed according to the manufacturer’s instructions and were quantified by absorbance at 492 nm at 24 and 48 h following the addition of 20 μM G7-peptide inhibitors.

### 4.4. Wound Healing Assay

MDA-MB-231 (2 × 10^5^ cells/well) or SKBR-3 (3 × 10^5^ cells/well) cells were seeded to 90% confluence in a 24-well plate for overnight culture. The following day a scratch was made through the center of each well using a 200-µL pipette tip, creating an open “wound” that was clear of cells. The dislodged cells were removed by two washes with complete culture media. 5 µg/mL of mitocycin-C (Sigma-Aldrich, St. Louis, MO, USA) was added throughout the experiments to prevent cell proliferation. Wound closure was monitored in real time using the Leica AF6000 LX live cell imaging system, and images were captured every 1 h over a 24 h (MDA-MB-231) or 48 h (SKBR-3) period. The normalized wound closure was calculated using the software TScratch (Version 1.0, CSElab, ETH Zurich, Switzerland; https://www.cse-lab.ethz.ch/software/) [58].

### 4.5. Transwell Motility Assay

MDA-MB-231 (6 × 10^4^ cells/well) or SKBR-3 (1 × 10^5^ cells/well) cells in 0.3 mL of serum-free medium were plated in the top chamber of the transwell with a noncoated polycarbonate membrane (6.5 mm diameter insert, 8.0 μm pore size; Corning Incorporated). The lower compartment was filled with normal culture medium, supplemented with 10% (*v/v*) FBS as a chemoattractant. After incubation for 4 h (MDA-MB-231) or 30 h (SKBR-3), cells on the lower surface of the membrane were fixed with 4% paraformaldehyde and stained with 0.2% crystal violet. Cells that did not migrate through the pores were mechanically removed by a cotton swab. The numbers of migrated cells were imaged using Olympus CKX41 inverted bright field microscope with color camera.

### 4.6. Transwell Invasion Assay

For the transwell invasion assay, cell culture inserts (8 µm pore, Corning Biocoat^TM^ Growth Factor Reduced MATRIGEL Invasion Chamber, BD Biosciences, San Jose, CA, USA) were rehydrated with warm serum-free medium for 2 h prior to the experiments. Cells were grown to 75% confluence and then starved for 24 h. 1.0 × 10^5^ cells were seeded in 0.5 mL serum-free medium in the upper chamber and the lower chamber was filled with 0.75 mL of growth medium with 10% (*v/v*) FBS as a chemoattractant. After 48 h incubation, invaded cells were fixed and visualized by staining with 0.2% crystal violet and counted using a 4× objective in each of three randomly chosen fields. Each experiment was performed in duplicate.

### 4.7. ITC Experiments

ITC experiments were performed on a MicroCal iTC200 with the cell temperature set at 25 °C, stirring speed of 750 rpm and a reference power of 10 μcal/sec. The lyophilised peptides were resuspended in buffer comprising 1 mM Na_3_PO_4_, 20 mM HEPES (pH 7.4), 150 mM NaCl and 4 mM β-mercaptoethanol. Grb7-SH2, produced as previously reported [21], was dialysed into the same buffer. Peptides were titrated into the sample cell over 20 injections of 2 μL, of 4 s durations with injection spacings of 180 s. The first injection was 0.5 μL over a 1 sec duration. If necessary, heats of dilution controls were determined by titrating the peptide into the sample cell containing buffer only, and the data subtracted from the Grb7-SH2/peptide thermograms. The corrected binding curve was fit by a single site binding model. Data were analyzed and plotted using NITPIC, SEDPHAT and GUSSI [59].

### 4.8. SPR Measurement of Lipid Bilayer Interactions

Peptide-membrane interactions were followed using an L1 biosensor chip at 25 °C in a Biacore 3000 instrument (GE healthcare, Uppsala, Sweden) using HEPES buffer (10 mM HEPES containing 150 mM NaCl, Ph 7.4) as running buffer. Synthetic lipids POPC and POPS were purchased from Avanti Polar Lipids (Alabastar, AL, USA) and were solubilized in chloroform and mixed in quantities required to form POPC/POPS (4:1) mixture. The lipids were resuspended in running buffer and sized by extrusion to form small unilamellar vesicles [60]. Vesicles were then deposited onto the L1 chip and confirmed to form stable lipid bilayers [60,61]. Peptide samples at varying concentrations (1–32 μM) were injected (180 s, 5 μL/min) over deposited lipid bilayers, and their dissociation was followed for 600 s. Chip surface was regenerated following protocols previously detailed [60]. Response unit were normalized to peptide-to-lipid ratio (P/L) as before [61].

### 4.9. Internalization of Peptide Inside Cells Quantified Using Mass Spectrometry

Adherent human negroid cervix epithelioid carcinoma cells (HeLa) were grown in DMEM (supplemented with 1% *w/v* penicillin/streptomycin and fetal bovine serum (10% *v/v*) and incubated in a humidified atmosphere (5% CO2, 37 °C). Internalization of peptide (20 μM) into HeLa cells was determined using mass spectrometry using a protocol previously detailed [41]. Briefly, cells were incubated with peptide for 1 h at 37 °C in medium without serum. Non-internalized peptide was removed and cells washed with PBS. Cells were harvested from the plate using trypsin, transferred to low-binding Eppendorf tubes, centrifuged (5000 g, 10 min at 4 °C), the supernantant removed and cells resuspended in PBS.

Multiple reaction monitoring (MRM) was used for the quantitative analysis of peptides. To distinguish between membrane-bound and soluble peptide, cell samples were disrupted and treated as previously described [41]. Peptide associated with cells and/or internalized was quantified via mass spectrometry in i) total cells (membrane-associated + in solution (i.e., cytosol or inside endosomes); ii) Pellet (membrane-associated); iii) in supernatant (i.e., cytosol or inside endosomes). Samples and standards were filtered with a 0.2 μm filter prior to mass spectrometric analysis. Following sample injection of 1 μL, a linear acetonitrile gradient was used to separate sample components via UPLC on a Kinetex C18 column of dimensions 100 × 2.1 mm with 1.7 μm particle size and 100 Å pore size (Phenomenex, Torrance, CA, USA) at a flow rate of 0.4 mL/min and analyte MRMs monitored using a QTRAP 6500+ LC-MS/MS system (AB/Sciex, Foster City, CA, USA) with source temperature set at 600 °C. Details of monitored transitions are provided in Supplementary Table S1, and illustrated in Supplementary Figure S2.

## Figures and Tables

**Table 1:** Thermodynamic parameters of Grb7 targeting peptides binding to the Grb7-SH2 ^#^.

Binding Parameter	G7-B7	G7-B7-Pen	G7-18NATE	G7-18NATE-Pen
*K*_b_ (10^6^ M^−1^)	2.06	5.95	1.00	0.290
Δ*H* (kcal·mol^−1^)	−16.0 (−15.8–−16.2)	−16.6 (−16.4–−17.0)	−12.7 (−12.5–−12.9)	−10.2 (−9.5–−10.9)
Δ*S* (kcal·mol^−1^·K^−1^)	−24.6	−24.8	−19.8	−9.19
−TΔ*S* (kcal·mol^−1^)	7.35	7.41	5.91	2.74
Δ*G* (kcal·mol^−1^)	−8.62	−9.24	−6.82	−7.45
*K*_D_ (μM)	0.48 (0.43–0.55)	0.17 (0.13–0.22)	10.1 (9.24–11.0)	3.45 (2.29–5.15)

^#^ The values in parentheses indicate a 95% confidence interval.

**Table 2:** Internalization and association of G7-peptides with HeLa cells.

Peptide	Total Concentration of Peptide Associated with Cells (nM)		Concentration of Peptide in Supernatant (nM)		Concentration of Peptide Associated with Pellet (nM)	
	Average	SEM	Average	SEM	Average	SEM
G7-18NATE	n.d.*	n.d.	n.d.	n.d.	n.d.	n.d.
G7-18NATE-Pen	696.4	112.2	221.1	49.2	312.8	60.8
G7-B7M2-Pen	208.0	12.7	n.d.	n.d.	86.7	36.1

* n.d., not detected.

**Table 3:** Percentage of unbound G7-peptides associated with HeLa cell structures.

Peptide	% Unbound Peptide Associated with Total Cell		% Unbound Peptide Associated with Supernatant		% Unbound Peptide Associated with Pellet	
	Average	SEM	Average	SEM	Average	SEM
G7-18NATE	n.d.*	n.d.	n.d.	n.d.	n.d.	n.d.
G7-18NATE-Pen	6.2%	1.0%	2.0%	0.4%	2.8%	0.5%
G7-B7M2-Pen	1.5%	0.1%	n.d.	n.d.	0.6%	0.3%

* n.d., not detected.

## Supplementary Materials

The following are available online. Table S1: Affinity of G7-peptides for Grb7-SH2 domain; Figure S1: Representative monolayer wound healing assays; Table S2: Peak areas for quantifier transitions; Figure S2: MRM plots.

## Abbreviations

Grb7	growth receptor bound protein
SH2 domain	Src homology domain 2
TNBC	triple negative breast cancer
cmF	carboxymethylphenylalanine
cF	carboxyphenylalanine
pY	phosphotyrosine
CPP	cell penetrating peptide
SPR	surface plasmon resonance
ITC	Isothermal titration calorimetry

## References

[1] Siveen K.S., Prabhu K.S., Achkar I.W., Kuttikrishnan S., Shyam S., Khan A.Q., Merhi M., Dermime S., Uddin S. (2018). Role of Non Receptor Tyrosine Kinases in Hematological Malignances and its Targeting by Natural Products. Mol. Cancer.

[2] Montor W.R., Salas A., Melo F.H.M. (2018). Receptor tyrosine kinases and downstream pathways as druggable targets for cancer treatment: The current arsenal of inhibitors. Mol. Cancer.

[3] Rimawi M.F., Schiff R., Osborne C.K. (2015). Targeting HER2 for the treatment of breast cancer. Annu. Rev. Med..

[4] Lucas-Fernandez E., Garcia-Palmero I., Villalobo A. (2008). Genomic organization and control of the Grb7 gene family. Curr. Genomics.

[5] Han D.C., Guan J.L. (1999). Association of focal adhesion kinase with Grb7 and its role in cell migration. J. Biol Chem..

[6] Han D.C., Shen T.L., Guan J.L. (2000). Role of Grb7 targeting to focal contacts and its phosphorylation by focal adhesion kinase in regulation of cell migration. J. Biol. Chem..

[7] Stein D., Wu J., Fuqua S.A., Roonprapunt C., Yajnik V., D′Eustachio P., Moskow J.J., Buchberg A.M., Osborne C.K., Margolis B. (1994). The SH2 domain protein GRB-7 is co-amplified, overexpressed and in a tight complex with HER2 in breast cancer. EMBO J..

[8] Nadler Y., Gonzalez A.M., Camp R.L., Rimm D.L., Kluger H.M., Kluger Y. (2010). Growth factor receptor-bound protein-7 (Grb7) as a prognostic marker and therapeutic target in breast cancer. Ann. Oncol..

[9] Ramsey B., Bai T., Hanlon Newell A., Troxell M., Park B., Olson S., Keenan E., Luoh S.W. (2011). GRB7 protein over-expression and clinical outcome in breast cancer. Breast Cancer Res. Treat..

[10] Bai T., Luoh S.W. (2007). GRB-7 facilitates HER-2/Neu-mediated signal transduction and tumor formation. Carcinogenesis.

[11] Chan D.W., Hui W.W., Cai P.C., Liu M.X., Yung M.M., Mak C.S., Leung T.H., Chan K.K., Ngan H.Y. (2012). Targeting GRB7/ERK/FOXM1 signaling pathway impairs aggressiveness of ovarian cancer cells. PLoS ONE.

[12] Pero S.C., Daly R.J., Krag D.N. (2003). Grb7-based molecular therapeutics in cancer. Expert Rev. Mol. Med..

[13] Chu P.Y., Tai Y.L., Shen T.L. (2019). Grb7, a Critical Mediator of EGFR/ErbB Signaling, in Cancer Development and as a Potential Therapeutic Target. Cells.

[14] Shen T.L., Guan J.L. (2004). Grb7 in intracellular signaling and its role in cell regulation. Front. Biosci..

[15] Han D.C., Shen T.L., Guan J.L. (2001). The Grb7 family proteins: Structure, interactions with other signaling molecules and potential cellular functions. Oncogene.

[16] Fiddes R.J., Campbell D.H., Janes P.W., Sivertsen S.P., Sasaki H., Wallasch C., Daly R.J. (1998). Analysis of Grb7 recruitment by heregulin-activated erbB receptors reveals a novel target selectivity for erbB3. J. Biol. Chem..

[17] Machida K., Mayer B.J. (2005). The SH2 domain: Versatile signaling module and pharmaceutical target. Biochim. Biophys. Acta.

[18] Morlacchi P., Robertson F.M., Klostergaard J., McMurray J.S. (2014). Targeting SH2 domains in breast cancer. Future Med. Chem..

[19] Pero S.C., Oligno L., Daly R.J., Soden A.L., Liu C., Roller P.P., Li P., Krag D.N. (2002). Identification of novel non-phosphorylated ligands, which bind selectively to the SH2 domain of Grb7. J. Biol. Chem..

[20] Gunzburg M.J., Ambaye N.D., Hertzog J.T., Borgo M.P., Pero S.C., Krag D.N., Wilce M.C.J., Aguilar M.I., Perlmutter P., Wilce J.A. (2010). Use of SPR to Study the Interaction of G7-18NATE Peptide with the Grb7-SH2 Domain. Int. J. Pept. Res. Ther..

[21] Gunzburg M.J., Ambaye N.D., Del Borgo M.P., Pero S.C., Krag D.N., Wilce M.C., Wilce J.A. (2012). Interaction of the non-phosphorylated peptide G7-18NATE with Grb7-SH2 domain requires phosphate for enhanced affinity and specificity. J. Mol. Recognit..

[22] Watson G.M., Lucas W.A.H., Gunzburg M.J., Wilce J.A. (2017). Insight into the Selectivity of the G7-18NATE Inhibitor Peptide for the Grb7-SH2 Domain Target. Front Mol. Biosci..

[23] Deshayes S., Morris M.C., Divita G., Heitz F. (2005). Cell-penetrating peptides: Tools for intracellular delivery of therapeutics. Cell Mol. Life Sci..

[24] Pradip D., Bouzyk M., Dey N., Leyland-Jones B. (2013). Dissecting GRB7-mediated signals for proliferation and migration in HER2 overexpressing breast tumor cells: GTP-ase rules. Am. J. Cancer Res..

[25] Giricz O., Calvo V., Pero S.C., Krag D.N., Sparano J.A., Kenny P.A. (2012). GRB7 is required for triple-negative breast cancer cell invasion and survival. Breast Cancer Res. Treat..

[26] Pero S.C., Shukla G.S., Cookson M.M., Flemer S., Krag D.N. (2007). Combination treatment with Grb7 peptide and Doxorubicin or Trastuzumab (Herceptin) results in cooperative cell growth inhibition in breast cancer cells. Br. J. Cancer.

[27] Tanaka S., Pero S.C., Taguchi K., Shimada M., Mori M., Krag D.N., Arii S. (2006). Specific peptide ligand for Grb7 signal transduction protein and pancreatic cancer metastasis. J. Natl. Cancer Inst..

[28] Ambaye N.D., Pero S.C., Gunzburg M.J., Yap M., Clayton D.J., Del Borgo M.P., Perlmutter P., Aguilar M.I., Shukla G.S., Peletskaya E. (2011). Structural basis of binding by cyclic nonphosphorylated peptide antagonists of Grb7 implicated in breast cancer progression. J. Mol. Biol..

[29] Watson G.M., Gunzburg M.J., Ambaye N.D., Lucas W.A., Traore D.A., Kulkarni K., Cergol K.M., Payne R.J., Panjikar S., Pero S.C. (2015). Cyclic peptides incorporating phosphotyrosine mimetics as potent and specific inhibitors of the Grb7 breast cancer target. J. Med. Chem..

[30] Gunzburg M.J., Ambaye N.D., Del Borgo M.P., Perlmutter P., Wilce J.A. (2013). Design and testing of bicyclic inhibitors of Grb7—Are two cycles better than one?. J. Pept. Sci..

[31] Gunzburg M.J., Kulkarni K., Watson G.M., Ambaye N.D., Del Borgo M.P., Brandt R., Pero S.C., Perlmutter P., Wilce M.C., Wilce J.A. (2016). Unexpected involvement of staple leads to redesign of selective bicyclic peptide inhibitor of Grb7. Sci. Rep..

[32] Watson G.M., Kulkarni K., Sang J., Ma X., Gunzburg M.J., Perlmutter P., Wilce M.C.J., Wilce J.A. (2017). Discovery, Development, and Cellular Delivery of Potent and Selective Bicyclic Peptide Inhibitors of Grb7 Cancer Target. J. Med. Chem..

[33] Lim R.C., Price J.T., Wilce J.A. (2014). Context-dependent role of Grb7 in HER2+ve and triple-negative breast cancer cell lines. Breast Cancer Res. Treat..

[34] Kalafatovic D., Giralt E. (2017). Cell-Penetrating Peptides: Design Strategies beyond Primary Structure and Amphipathicity. Molecules.

[35] Maiolo J.R., Ferrer M., Ottinger E.A. (2005). Effects of cargo molecules on the cellular uptake of arginine-rich cell-penetrating peptides. Biochim. Biophys. Acta.

[36] Oba M., Kato T., Furukawa K., Tanaka M. (2016). A Cell-Penetrating Peptide with a Guanidinylethyl Amine Structure Directed to Gene Delivery. Sci. Rep..

[37] Watson G.M., Kulkarni K., Brandt R., Del Borgo M.P., Aguilar M.I., Wilce J.A. (2017). Shortened Penetratin Cell-Penetrating Peptide Is Insufficient for Cytosolic Delivery of a Grb7 Targeting Peptide. ACS Omega.

[38] Hedegaard S.F., Derbas M.S., Lind T.K., Kasimova M.R., Christensen M.V., Michaelsen M.H., Campbell R.A., Jorgensen L., Franzyk H., Cardenas M. (2018). Fluorophore labeling of a cell-penetrating peptide significantly alters the mode and degree of biomembrane interaction. Sci. Rep..

[39] Birch D., Christensen M.V., Staerk D., Franzyk H., Nielsen H.M. (2017). Fluorophore labeling of a cell-penetrating peptide induces differential effects on its cellular distribution and affects cell viability. Biochim. Biophys. Acta.

[40] Kauffman W.B., Fuselier T., He J., Wimley W.C. (2015). Mechanism Matters: A Taxonomy of Cell Penetrating Peptides. Trends Biochem. Sci..

[41] Henriques S.T., Huang Y.H., Chaousis S., Sani M.A., Poth A.G., Separovic F., Craik D.J. (2015). The Prototypic Cyclotide Kalata B1 Has a Unique Mechanism of Entering Cells. Chem. Biol..

[42] Hall K., Lee T.H., Aguilar M.I. (2011). The role of electrostatic interactions in the membrane binding of melittin. J. Mol. Recognit..

[43] Cascales L., Henriques S.T., Kerr M.C., Huang Y.H., Sweet M.J., Daly N.L., Craik D.J. (2011). Identification and characterization of a new family of cell-penetrating peptides: Cyclic cell-penetrating peptides. J. Biol. Chem..

[44] Henriques S.T., Melo M.N., Castanho M.A. (2006). Cell-penetrating peptides and antimicrobial peptides: How different are they?. Biochem. J..

[45] Watson H. (2015). Biological membranes. Essays Biochem..

[46] Ingolfsson H.I., Melo M.N., van Eerden F.J., Arnarez C., Lopez C.A., Wassenaar T.A., Periole X., de Vries A.H., Tieleman D.P., Marrink S.J. (2014). Lipid organization of the plasma membrane. J. Am. Chem. Soc..

[47] van Meer G., Voelker D.R., Feigenson G.W. (2008). Membrane lipids: Where they are and how they behave. Nat. Rev. Mol. Cell Biol..

[48] Utsugi T., Schroit A.J., Connor J., Bucana C.D., Fidler I.J. (1991). Elevated expression of phosphatidylserine in the outer membrane leaflet of human tumor cells and recognition by activated human blood monocytes. Cancer Res..

[49] Zwaal R.F., Comfurius P., Bevers E.M. (2005). Surface exposure of phosphatidylserine in pathological cells. Cell Mol. Life Sci..

[50] El-Andaloussi S., Jarver P., Johansson H.J., Langel U. (2007). Cargo-dependent cytotoxicity and delivery efficacy of cell-penetrating peptides: A comparative study. Biochem. J..

[51] Chu P.Y., Li T.K., Ding S.T., Lai I.R., Shen T.L. (2010). EGF-induced Grb7 recruits and promotes Ras activity essential for the tumorigenicity of Sk-Br3 breast cancer cells. J. Biol. Chem..

[52] Al Soraj M., He L., Peynshaert K., Cousaert J., Vercauteren D., Braeckmans K., De Smedt S.C., Jones A.T. (2012). siRNA and pharmacological inhibition of endocytic pathways to characterize the differential role of macropinocytosis and the actin cytoskeleton on cellular uptake of dextran and cationic cell penetrating peptides octaarginine (R8) and HIV-Tat. J. Controlled Release.

[53] Rhodes C.A., Pei D. (2017). Bicyclic Peptides as Next-Generation Therapeutics. Chemistry.

[54] Lian W., Jiang B., Qian Z., Pei D. (2014). Cell-permeable bicyclic peptide inhibitors against intracellular proteins. J. Am. Chem. Soc..

[55] Yau W.M., Wimley W.C., Gawrisch K., White S.H. (1998). The preference of tryptophan for membrane interfaces. Biochemistry.

[56] Habault J., Poyet J.L. (2019). Recent Advances in Cell Penetrating Peptide-Based Anticancer Therapies. Molecules.

[57] Ramaker K., Henkel M., Krause T., Rockendorf N., Frey A. (2018). Cell penetrating peptides: A comparative transport analysis for 474 sequence motifs. Drug Deliv..

[58] Geback T., Schulz M.M., Koumoutsakos P., Detmar M. (2009). TScratch: A novel and simple software tool for automated analysis of monolayer wound healing assays. Biotechniques.

[59] Brautigam C.A., Zhao H., Vargas C., Keller S., Schuck P. (2016). Integration and global analysis of isothermal titration calorimetry data for studying macromolecular interactions. Nat. Protoc..

[60] Henriques S.T., Pattenden L.K., Aguilar M.I., Castanho M.A. (2008). PrP(106–126) does not interact with membranes under physiological conditions. Biophys. J..

[61] Henriques S.T., Huang Y.H., Castanho M.A., Bagatolli L.A., Sonza S., Tachedjian G., Daly N.L., Craik D.J. (2012). Phosphatidylethanolamine binding is a conserved feature of cyclotide-membrane interactions. J. Biol. Chem..

