# Supplementary material for: Evaluation of Cyclic Peptide Inhibitors of the Grb7 Breast Cancer Target: Small Change in Cargo Results in Large Change in Cellular Activity
Source: Molecules. 2019 Oct 17;24(20):3739. doi: 10.3390/molecules24203739 (PMC6832895; doi:10.3390/molecules24203739)

Supplementary Materials

Evaluation of Cyclic Peptide Inhibitors of the Grb7 Breast Cancer Target: Small Change in Cargo Results in Large Change in Cellular Activity

Jianrong Sang [1,2], Ketav Kulkarni [2], Gabrielle M. Watson [2], Xiuquan Ma [2], David J. Craik [3], Sónia T. Henriques [3,4], Aaron G. Poth [3], Aurélie H. Benfield [3,4] and Jacqueline A. Wilce [2,*]

[1] Department of Physiology, School of Medicine, Jiangsu University, 301 Xuefu Road, Zhenjiang, Jiangsu 212013, China; sangjianrong@ujs.edu.cn (J.S.)

[2] Biomedicine Discovery Institute, Department of Biochemistry and Molecular Biology, Monash University, Wellington Road, 3800 Clayton, Australia; ketav.kulkarni@monash.edu (K.K.); gabrielle.watson@monash.edu (G.M.W.); hugh.ma@monash.edu (X.M.)

[3] Institute for Molecular Bioscience, The University of Queensland, 4072 Brisbane, Australia; d.craik@imb.uq.edu.au (D.J.C.); sonia.henriques@qut.edu.au (S.T.H.); a.poth@imb.uq.edu.au (A.G.P.); aurelie.benfield@qut.edu.au (A.H.B.)

[4] School of Biomedical Sciences, Institute of Health & Biomedical Innovation, Queensland University of Technology, Translational Research Institute, 4102 Brisbane, Australia

* Correspondence: jackie.wilce@monash.edu; Tel. +613-9902-9226; Fax: +613-9902-9500

Supporting Information Table S1

Affinity of G7-peptides for Grb7-SH2 domain

Provided is a summary of G7-peptides previously reported. The schematics show the single letter amino acid nomenclature in coloured font and chemical structures are represented in black.

Peptide Name	K_D (μM)	Buffer	Reference
G7-18NATE<br>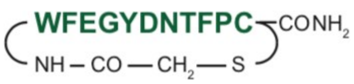	18.1	1 mM Na_3PO_4, 20 mM Tris, 150 mM NaCl, 1 mM DTT (pH 7.4)	[1]
G7-M1<br>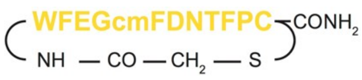	5.7	1 mM Na_3PO_4, 20 mM Tris, 150 mM NaCl, 1 mM DTT (pH 7.4)	[1]
G7-M2<br>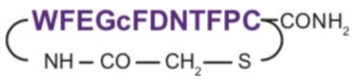	2.1	1 mM Na_3PO_4, 20 mM Tris, 150 mM NaCl, 1 mM DTT (pH 7.4)	[1]
G7-B1<br>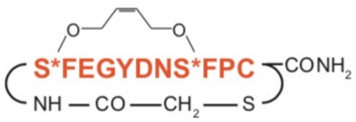	1.5	50 mM Na_3PO4, 150 mM NaCl, 1 mM DTT (pH 7.4)	[2]
G7-B4<br>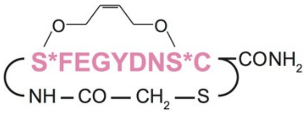	0.83	50 mM Na_3PO4, 150 mM NaCl, 1 mM DTT (pH 7.4)	[2]
G7-B5<br>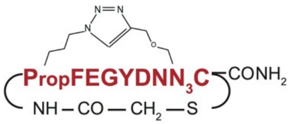	7.8	1 mM Na_3PO4, 20 mM Tris, 150 mM NaCl, 1 mM DTT (pH 7.4)	[3]
G7-B6<br>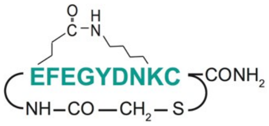	15.7	1 mM Na_3PO4, 20 mM Tris, 150 mM NaCl, 1 mM DTT (pH 7.4)	[3]

G7-B7 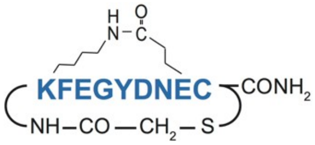	1.1	1 mM Na_3PO4, 20 mM Tris, 150 mM NaCl, 1 mM DTT (pH 7.4)	[3]
G7-B7M1 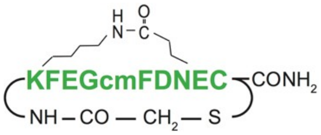	0.22	1 mM Na_3PO4, 20 mM Tris, 150 mM NaCl, 1 mM DTT (pH 7.4)	[3]
G7-B7M2 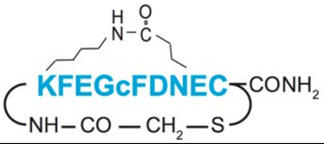	0.13	1 mM Na_3PO4, 20 mM Tris, 150 mM NaCl, 1 mM DTT (pH 7.4)	[3]

References

1. Watson, G.M.; Gunzburg, M.J.; Ambaye, N.D.; Lucas, W.A.; Traore, D.A.; Kulkarni, K.; Cergol, K.M.; Payne, R.J.; Panjikar, S.; Pero, S.C., et al. Cyclic peptides incorporating phosphotyrosine mimetics as potent and specific inhibitors of the Grb7 breast cancer target. *J. Med. Chem.* **2015**, *58*, 7707-7718.
2. Gunzburg, M.J.; Kulkarni, K.; Watson, G.M.; Ambaye, N.D.; Del Borgo, M.P.; Brandt, R.; Pero, S.C.; Perlmutter, P.; Wilce, M.C.; Wilce, J.A. Unexpected involvement of staple leads to redesign of selective bicyclic peptide inhibitor of Grb7. *Sci. Rep.* **2016**, *6*, 27060.
3. Watson, G.M.; Kulkarni, K.; Sang, J.; Ma, X.; Gunzburg, M.J.; Perlmutter, P.; Wilce, M.C.J.; Wilce, J.A. Discovery, Development, and Cellular Delivery of Potent and Selective Bicyclic Peptide Inhibitors of Grb7 Cancer Target. *J Med Chem* **2017**, *60*, 9349-9359.

Supporting Information Figure S1

Representative monolayer wound healing assays conducted for SKBR-3 (left) and MDA-MB-231 cells (right) treated with 1: control; 2: Pen; 3: G7-B7-Pen; 4: G7-B7M2-Pen; 5: G7-M2-Pen; 6: G7-18NATE-Pen. Results of triplicate experiments form the basis of Figure 4 of the main manuscript.

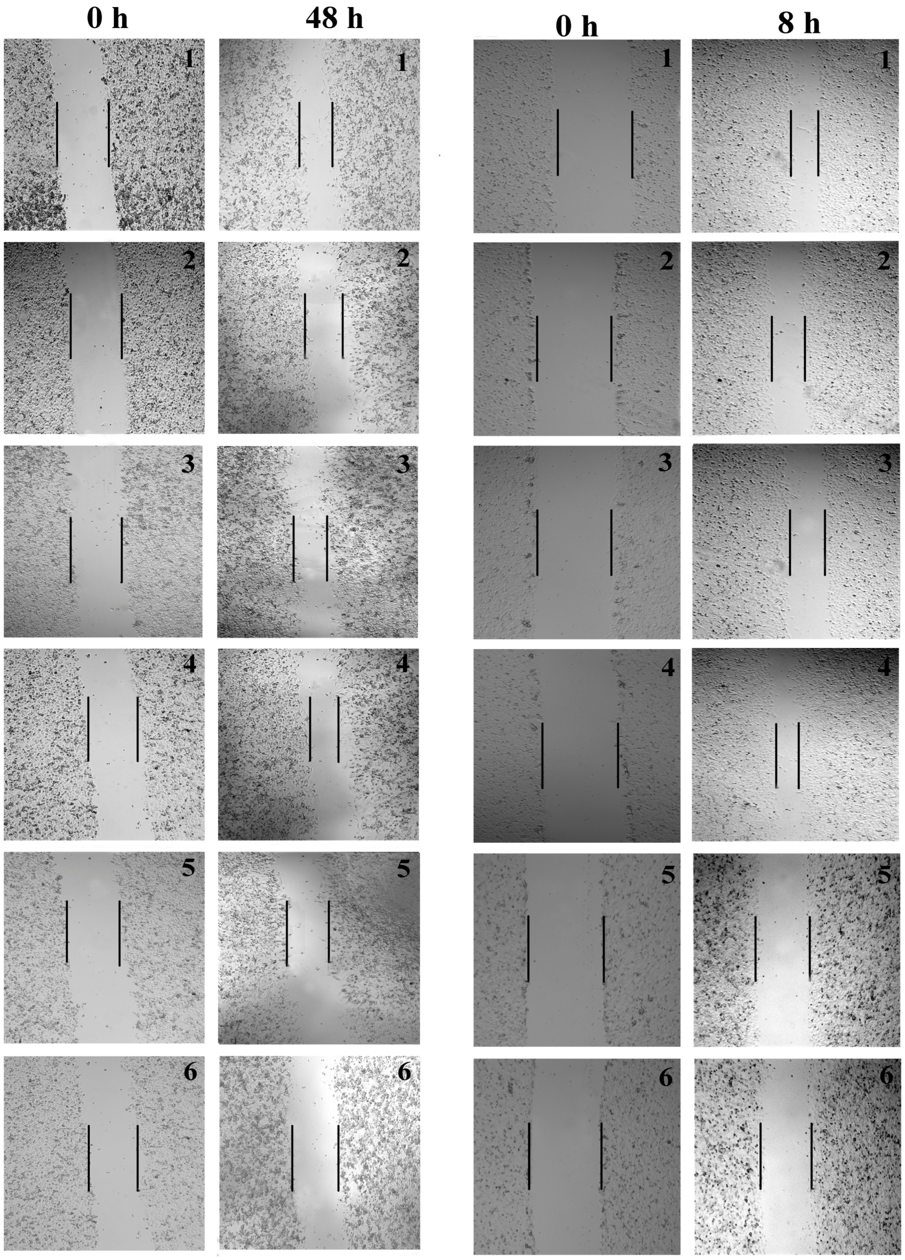

Supporting Information Table S2

Peak areas for quantifier transitions were normalized against internal standard using MultiQuant v3.0 (SCIEX) software. Peptide concentration in each sample was determined via comparison of normalized signal peak areas with external calibration curves made in sample-equivalent matrices on the same day as the cell assay. The experiment was performed in triplicate and repeated on two independent days.

Analyte	Transition	Q1 (*m/z*)	Q3 (*m/z*)	CE[a] (V)	DP[b] (V)	RT (mins)
G7-18NATE	Quantifier	720.3	1077.2	31	130	2.44
"	Qualifier	720.3	1264.3	40	130	2.44
"	Qualifier	720.3	1094.3	41	130	2.44
G7-18NATE-Pen	Quantifier	911.9	907.9	43	130	1.91
"	Qualifier	1215.6	1210.1	66	130	1.91
"	Qualifier	911.9	1095.1	51	130	1.91
G7-B7M2-Pen	Quantifier	677.1	798.1	31	130	1.66
"	Qualifier	846.1	1079.0	46	130	1.66
"	Qualifier	846.1	1035.6	47	130	1.66

[a] Collision energy; [b] declustering potential.

Reference

Anderson, L., and Hunter, C. L. (2006) Quantitative Mass Spectrometric Multiple Reaction Monitoring Assays for Major Plasma Proteins. Mol. Cell. Proteom. 5, 573-588.

Supporting Information Figure S2

MRM plots for G7-18NATE (panel A), G7-18NATE-Pen (panel B), and G7-B7M2-Pen (panel C), illustrating coelution and relative intensities of their transitions. MRM quantifier shown with blue trace and (blue) and qualifiers in (green and red).

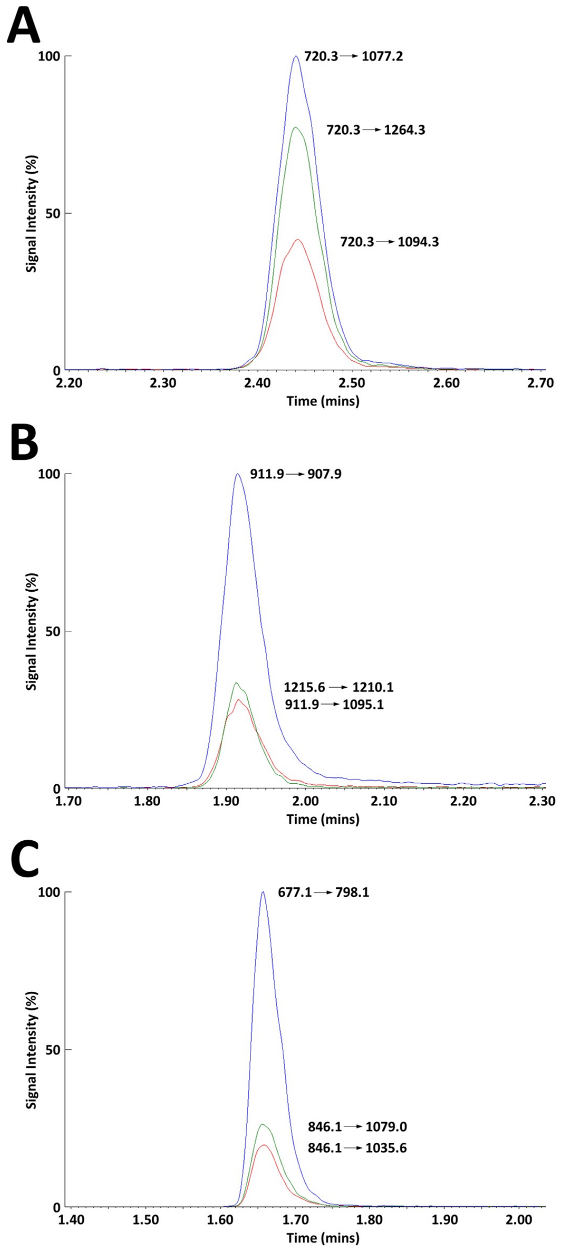

Supplement: Supplementary file 1 [file molecules-24-03739-s001.pdf]
